# Efficient Removal of Nonylphenol Isomers from Water by Use of Organo-Hydrotalcites

**DOI:** 10.3390/ijerph19127214

**Published:** 2022-06-12

**Authors:** Daniel Cosano, Dolores Esquivel, Francisco J. Romero-Salguero, César Jiménez-Sanchidrián, José Rafael Ruiz

**Affiliations:** Departamento de Química Orgánica, Instituto Universitario de Investigación en Química Fina y Nanoquímica IUNAN, Facultad de Ciencias, Universidad de Córdoba, Campus de Rabanales, Edificio Marie Curie, E-14071 Córdoba, Spain; q12esmem@uco.es (D.E.); qo2rosaf@uco.es (F.J.R.-S.); qo1jisac@uco.es (C.J.-S.)

**Keywords:** hydrotalcite, deoxycholate, adsorption, nonylphenol removal

## Abstract

The presence of potent organic endocrine-disrupting chemicals (EDCs) in natural aquifers can have adverse impacts on public health and the environment. 4-nonylphenol, one such EDC, can be efficiently removed from water by adsorption onto a clayey material. In this work, we created an effective sorbent for this purpose by using co-precipitation and subsequent ion-exchange to intercalate the organic anion deoxycholate into a Mg/Al hydrotalcite. Intercalating deoxycholate ions increased the organophilicity of the hydrotalcite surface. The solid was used to adsorb 4-nonylphenol at different pollutant concentrations and temperatures. The adsorption process was subjected to a kinetic study. Based on the results, the EDC was adsorbed by chemisorption. In addition, based on the equilibrium isotherms used for the process, the Freundlich model was the most accurate in reproducing the adsorption of 4-nonylphenol onto deoxycholate-intercalated hydrotalcite.

## 1. Introduction

**Figure 1:**
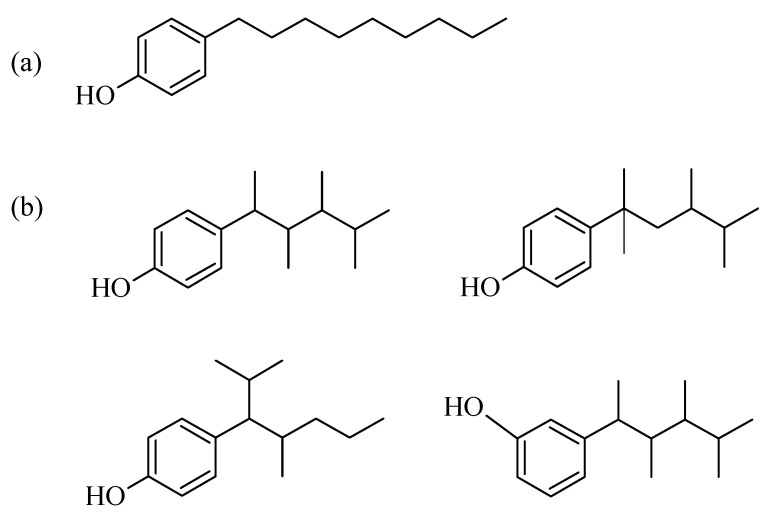
Chemical structure of 4-nonylphenol (4-NPhOH) (**a**); chain and position isomers of nonylphenol (**b**).

An increasing number of aquifers are being contaminated with endocrine-disrupting chemicals (EDCs). Although EDC concentrations in water are usually very low (typically in the microgram-per-liter or nanogram-per-liter range [1,2]), they can have deleterious effects on human health, such as endocrine disorders or abnormal organ development [3,4,5]. The presence of EDCs in water usually results from their use as additives in detergents, perfumes, and creams, among other products. Because most wastewater purification plants fail to remove the typically low concentrations of EDCs [6], these organic compounds must be eliminated with alternative procedures such as chemical oxidation [7] or physisorption [8,9,10,11]. Worthy of note is that among EDCs are nonylphenols, which typically comprise a mixture of chain and position isomers (NPhOH) and linear polyphenol at position 4 on an aromatic ring (4-NPhOH; Figure 1). These compounds, and short-chain ethoxylated nonylphenols, are usually degradation products of long-chain ethoxylated nonylphenols, which are surface-active and used in a variety of detergent, pesticide, packaging plastic, and cosmetic formulations. Upon discharge into the environment, these products are microbially converted into nonylphenols (particularly 4-NPhOH, which is the most widely used by the detergent industry [12]).

One of the main reasons for removing nonylphenols is their usually high concentration found in sewage sludge—a result of the ease with which they can be adsorbed onto solids such as sediments, sludge, and soil. Using sewage sludge in agriculture [13] can lead to contamination not only of soil, crops, and living organisms, but also of underground waters by leaching and ground waters by run-off [14]. Due to the high affinity of nonylphenol for some solids, a variety of materials have been used to remove nonylphenols from water by adsorption, including mica [8], organic polymers [9,15], modified clays [16], iron oxides [17,18], carbon nanotubes [19], and graphene oxides [20,21].

Hydrotalcites, also known as “anionic clays” or “layered double hydroxides”, are widely used as sorbents for both organic and inorganic water pollutants [22,23,24,25]. Hydrotalcite, the parent compound, is a natural mineral of the formula Mg_6_Al_2_(CO_3_)_2_(OH)_2_·6H_2_O, where magnesium and aluminum can be substituted by another divalent or tervalent metal, respectively, provided its ionic radius is similar to that of Mg or Al, respectively. In addition, carbonate ion can be substituted by a wide range of organic and inorganic anions [26,27]. The general formula for a hydrotalcite is thus [*M*(II)_1−*x*_*M*(III)*_x_*(OH)_2_]*^x^*^+^[*A_n_*_/*x*_]*^n^*^−^·*m*H_2_O, where *M*(II) is a divalent metal, *M*(III) is a tervalent one, *A* is an anion, *x* is the atomic ratio *M*(III)/[*M*(II) + *M*(III)], and *m* is the number of water molecules present in the interlayer region. Usually, *x* can range from 0.2 with a metal ratio of 4 to 0.33 with 1 of 2.

Structurally, hydrotalcites are similar to the natural magnesium oxide brucite [Mg(OH)_2_]. Thus, they consist of positively charged octahedral layers of metal hydroxides intercalated with anions that offset the positive charge. The interlayer region can additionally accommodate water molecules forming hydrogen bonds with the anion and/or metal layers.

Our research group previously used hydrotalcites as sorbents for cyanide [28]. However, despite their wide use as sorbents, these solids have very rarely, to our knowledge, been used to remove nonylphenol [29,30]. In this work, a hydrotalcite intercalated with an organic anion (deoxycholate) was, for the first time, used as a sorbent for nonylphenol in water. Organo-HT appears to be a suitable alternative to the adsorption, with calcined hydrotalcites easing the reusability of the material.

## 2. Materials and Methods

### 2.1. Materials

Sodium deoxycholate (DCH) and nonylphenol (Figure 1) were Sigma–Aldrich (USA) ref. D6750 and 290858. The metal salts [Mg(NO_3_)_2_·6H_2_O, ref. 141402; Al(NO_3_)_3_·9H_2_O, ref. 131099] and sodium hydroxide (NaOH, ref. 141687) were supplied by Panreac.

### 2.2. Preparation of the Sorbent

The sorbent used was a hydrotalcite with a Mg/Al ratio of 2.5 and containing intercalated deoxycholate anion to counter-charge in the metal layers. The solid was obtained in two steps. The first involved using a co-precipitation method to synthetize a Mg/Al hydrotalcite with nitrate as an interlayer anion, and the second involved exchanging the nitrate with deoxycholate ion by using a microwave-assisted procedure previously developed by our group [31]. Compositional and structural properties of the resulting hydrotalcite, designated HT-DSC, are summarized in Table 1.

### 2.3. Adsorption Tests

The adsorption of 4-nonylphenol (4-NPhOH) onto deoxycholate-intercalated hydrotalcite was examined by using solutions containing variable concentrations of adsorbate at their natural pH at room temperature. Each solution was prepared by adding an appropriate amount of 4-NPhOH to a 250 mL flask containing 25 mL of distilled water and then 100 mg of HT-DSC under continuous stirring, with periodic withdrawal of samples to quantify the amount of 4-NPhOH remaining in solution by gas chromatography–mass spectrometry (GC–MS). Once adsorption equilibrium was reached, stirring of the solution was continued for a few hours before it was stopped and the solid filtered off for GC–MS analysis on a Varian 3900 chromatograph coupled to a Varian Saturn 2100T mass spectrometer.

### 2.4. Kinetic Tests

One of the central aspects of an adsorption process is its kinetics. The kinetics of 4-nonylphenol adsorption onto the hydrotalcite was examined by using the previous 25 mL solution under the conditions described in Section 2.2. The amount of 4-NPhOH adsorbed onto the solid was calculated from the equation *q_t_* = ([NPhOH]_0_ − [NPhOH]*_t_*/*m*)·*V*, where *q_t_* is the adsorption capacity of the hydrotalcite at time *t* in µg 4-NPhOH/g hydrotalcite; *V* is the volume of solution in mL; and [NPhOH]_0_ and [NPhOH]*_t_* are the initial concentrations of adsorbate at time *t* in ppm. The optimum amounts of 4-NPhOH and sorbent, and the optimum temperature, to be used were all determined by testing.

The isotherms for nonylphenol adsorption onto the hydrotalcite were obtained with the batch equilibrium method, changing the concentration of nonylphenol and keeping all other variables constant over a long enough period for equilibrium to be reached.

## 3. Results and Discussion

### 3.1. Adsorption Tests

**Figure 2:**
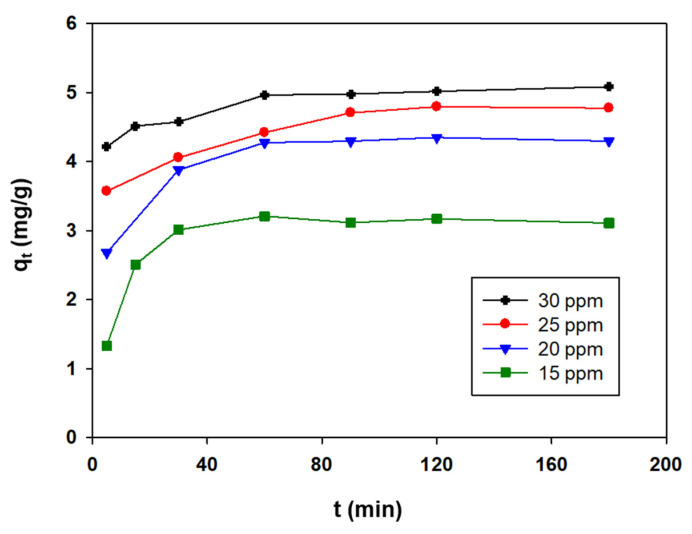
Influence of the initial concentration on the adsorption of 4-NPhOH on HT-DSC (experimental conditions: 25 mL of solution; 100 mg of adsorbent; 24 °C).

Figure 2 shows the time course of the 4-NPhOH concentration in the adsorption tests. Equilibrium was reached after about 60 min. The curve initially exhibited a very steep slope owing to the large number of free adsorption sites present in the hydrotalcite. However, the rate of the process decreased as such sites were gradually occupied by the adsorbate until equilibrium was reached. As can be seen from Figure 2, the lower the initial concentration of nonylphenol, the lower at which the system equilibrated. This was a logical result as the amount of sorbent used was identical in all tests, so the ratio of sorbent sites to nonylphenol molecules increased with increasing concentration of adsorbate.

The initially fast adsorption of 4-NPhOH was a result of the high capacity of its molecules to interact with deoxycholate ions on the HT-DSC surface. Moreover, the decrease in adsorption rate apparent from Figure 2 must have resulted from 4-NPhOH diffusing over the interlayer region in the sorbent. Therefore, 4-NPhOH was adsorbed onto the outer surface of the hydrotalcite at a high rate. After the surface saturated with adsorbate, excess 4-NPhOH started to diffuse over the hydrotalcite pore network and was adsorbed on the interlayer region [32], all at a much lower rate than in the previous process. As can be seen from the curves of Figure 2, the initial concentration of 4-NPhOH was strongly influential on its adsorption. Increasing the number of 4-NPhOH molecules in the solution increased the rate of mass transfer—and hence that of adsorption onto the hydrotalcite surface [32]—and the amount of nonylphenol adsorbed as a result.

**Figure 3:**
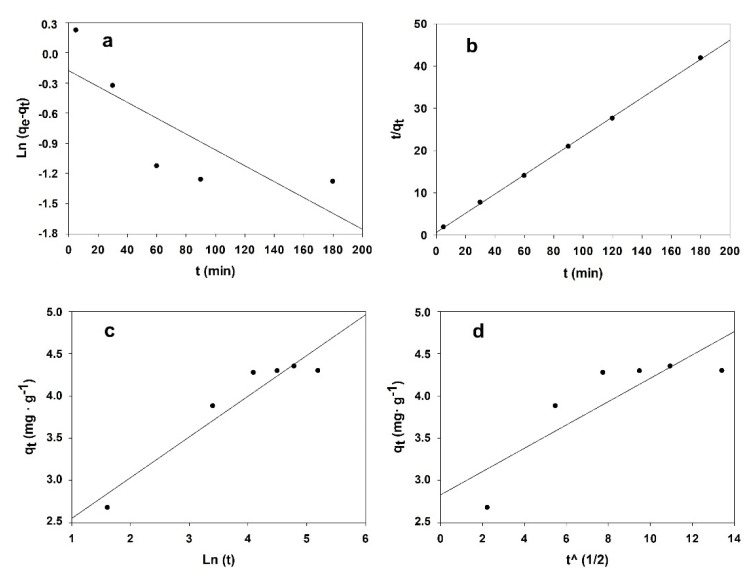
Kinetic models for 20 ppm concentration: (**a**) pseudo-first order kinetic model; (**b**) pseudo-second order kinetic model; (**c**) Elovich kinetic model; (**d**) and interparticle diffusion kinetic model.

Several kinetics models, represented below, were used to illustrate the 4-NPhOH adsorption into the HT-DSC (see Figure 3 and Figures S1–S3) [33,34]. It is essential to be wary that a relatively high R^2^ value for a specific kinetic model does not incidentally mean that this model is the only descriptive model [34,35,36,37].

The adsorption process was examined in the light of a pseudo-first order model and a pseudo-second order model. The former uses eq. 1 to represent the rate of adsorption of a liquid phase onto a solid phase.

log (*q*_e_ − *q_t_*) = log *q*_e_ − *k*_1_·*t,* (1)

where *q*_e_ and *q_t_* are the amounts of 4-NPhOH adsorbed per gram of hydrotalcite at equilibrium and time *t*, respectively, and *k*_1_ is the pseudo-first-order kinetic constant for the process.

The pseudo-second order model is based on Equation (2) and assumes that the adsorbate is adsorbed by chemisorption.

t/*q_t_* = (1/*k*_2_·*q*_e_^2^) + (*t*/*q*_e_)*,* (2)

where *q*_e_ and *q_t_* have the same meaning as in Equation (1), and *k*_2_ is the pseudo-second order rate constant for the process.

Table 2 shows the kinetic parameters and correlation coefficients obtained within each model. As can be seen, the pseudo-second order model provided better correlation. This suggests that, as shown below, 4-NPhOH was chemisorbed onto the hydrophobic, hydrocarbon portion of deoxycholate on the outer surface of the hydrotalcite.

The kinetic study was expanded by using the Elovich equation, which is typically used to describe the kinetics of a pseudo-second order process when its steady-state energy is not constant and the adsorbate is chemically adsorbed:

*q_t_* = (Ln *h*_b_ + Ln *t*)/*B,* (3)

where *h*_b_ and *B* are Elovich’s constants.

As can be inferred from Figure 3c, nonylphenol was adsorbed in two steps, namely: a very fast step where most of the adsorbate was adsorbed onto the hydrotalcite, followed by a slower step where the remainder adsorbate was incorporated onto the sorbent.

As confirmed by the intraparticle diffusion model, the adsorption process was chemical in nature. The hypothesis of intraparticle diffusion within pores in a sorbent particle assumes that the adsorbate is carried through the inner structure of the sorbent pore network by diffusion. Intraparticle diffusion conforms to eq. 4, which relates to specific adsorption to the square root of time.

*q_t_* = (*k*_intra_·*t*^0.5^) + *C,* (4)

where *C* is the concentration of adsorbate initially adsorbed.

We also examined the influence of temperature on the adsorption process. Some authors have shown that temperature strongly influences the adsorption of organic compounds onto various types of solid sorbents [9,19,35,38]. In this work, we examined the adsorption of 4-NPhOH onto HT-DSC at 298, 313, and 333 K. The rate of 4-NPhOH adsorption onto the hydrotalcite was found to increase with increasing temperature (results not shown); therefore, the process was endothermic. The activation energy was calculated from the Arrhenius equation in logarithmic form:

Ln *k*_2_ = ln *A* − (*E*_a_/R*T*), (5)

**Figure 4:**
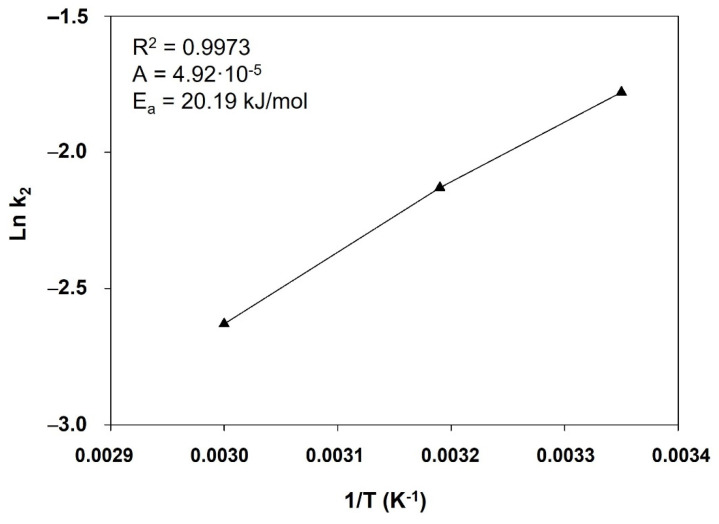
Variation of ln k_2_ versus the inverse of temperature.

A plot of ln *k*_2_ against the reciprocal temperature (Figure 4) was a straight line with a very high correlation coefficient that allowed the Arrhenius constant and activation energy to be easily calculated. The latter was 20.19 kJ/mol. This shows that the adsorption process was by chemical adsorption [39].

**Figure 5:**
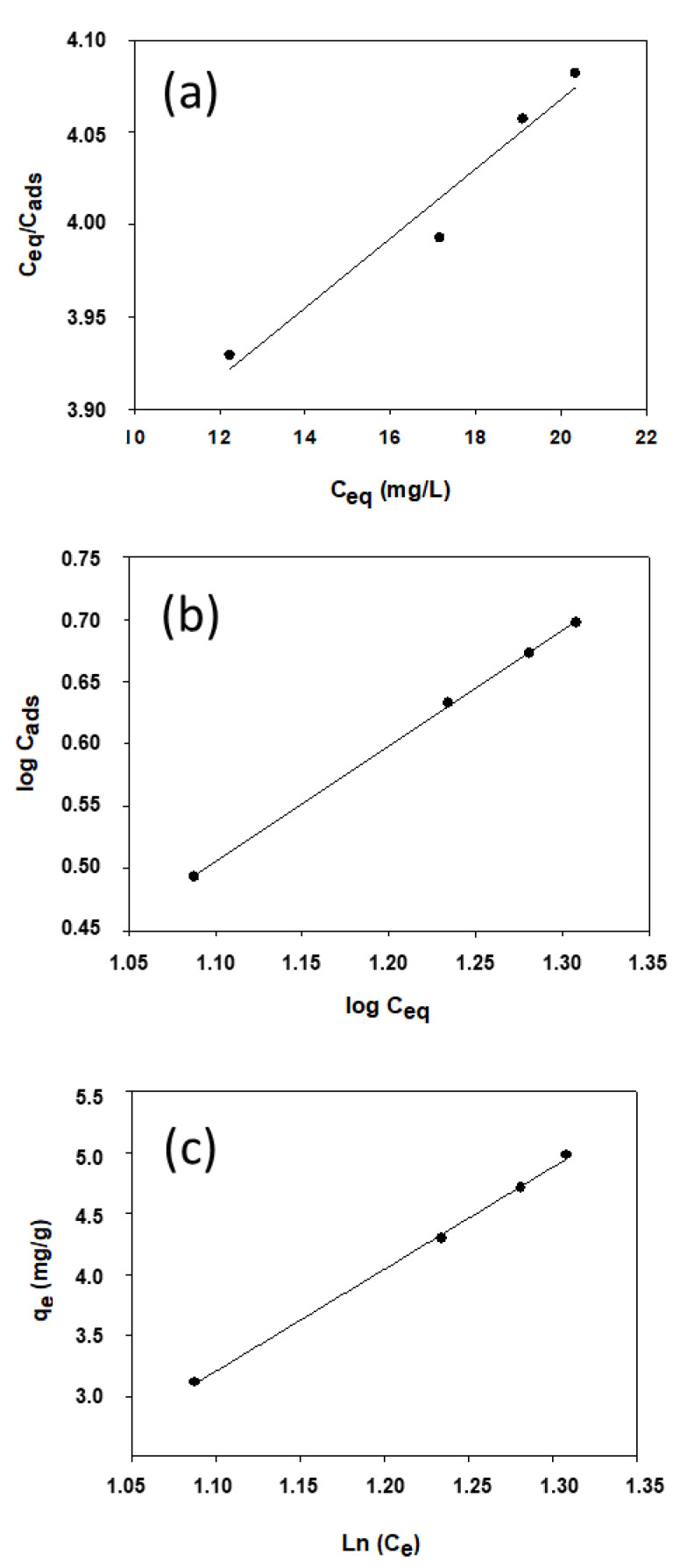
Langmuir (**a**), Freundlich (**b**), and Temkin (**c**) plots for 4-NPhOH adsorption on the HT-DSC.

The adsorption isotherm was constructed from the results obtained at 298 K. The experimental data were fitted by using the three most common models for this purpose, which are based on the Langmuir, Freundlich, and Temkin isotherms (see Figure 5) [34,40].

The Langmuir isotherm is a theoretical model accurately representing monolayer adsorption onto a uniform surface containing a finite number of specific adsorption sites, where the adsorbate and sorbent interact to a negligible extent. Mathematically, the Langmuir isotherm is defined by

*C*_eq_/*C*_ads_ = (1/*bQ*) + (*C*_eq_/*Q*), (6)

where *C*_eq_ is the equilibrium adsorption concentration of 4-NPhOH (mg/L), *Q* is the amount of 4-NPhOH adsorbed per unit mass of sorbent (mg/g) with monolayer coverage, *C*_ads_ is the *Q* value at equilibrium (mg/g), and *b* is a constant related to the affinity of binding sites.

The Freundlich model is based on an empirical equation assuming energy non-uniformity among specific adsorption sites, and exposes a consistently exponential distribution of active sites over a non-uniform surface:

*C*_ads_ = *K*_f_·*C*_eq_^1/*n*^, (7)

Or, in logarithmic form,

log *C*_ads_ = log *K*_f_ + (1/*n*)·log *C*_eq_, (8)

where *K*_f_ and *n* are two Freundlich temperature-dependent constants.

The Temkin model assumes a uniform distribution of binding energies and introduces constants depending on the initial heat of adsorption. It also assumes such heat to decrease linearly with increasing coverage. The model is based on the following equation:

*q*_e_ = R*T*·(Ln *K_T_* + Ln *C*_e_)/*b_T_*, (9)

where *K_T_* and *b_T_* are Temkin’s equilibrium binding constant (L/g) and adsorption heat constant, respectively.

Table 3 shows the figures of merit of each type of isotherm and its correlation coefficients (*R*^2^). The results suggest that the Freundlich model describes the sorption equilibrium more accurately than the Langmuir and Temkin models. Thus, 4-NPhOH adsorption on the hydrotalcite does not result in complete coverage of the solid surface; the adsorption energy decreases exponentially as 4-NPhOH is adsorbed onto the solid. As can be seen from Table 3, the Temkin model exhibited high correlation, which is unsurprising since it assumes the adsorption energy to decrease linearly with increasing adsorption of the adsorbate—as it did here based on the results obtained with the Freundlich model. In general, the hydrophobic interaction was the main sorption mechanism when both organic pollutants and the surface of materials are hydrophobic.

R_L_ is the dimensionless constant that predicts whether the adsorption system is efficient or not, defined by Weber and Chakravorti [41] as:

R_L_ =1/(1 + K_L_ · C_0_), (10)

where K_L_ is the constant of Langmuir and C_0_ is intimal 4-NPhOH concentration (mg*g^−1^). The parameter R_L_ indicates the shape of isotherm, where: 0 < RL < 1 the adsorption is favorable, RL > 1 the adsorption is unfavorable, R_L_ = 1 is linear adsorption and R_L_ = 0 is irreversible [42,43]. For 4-NPhOH, a value of R_L_ = 0.3378 is obtained, which means the adsorption process is favorable.

### 3.2. Reusability of the Adsorbent

**Figure 6:**
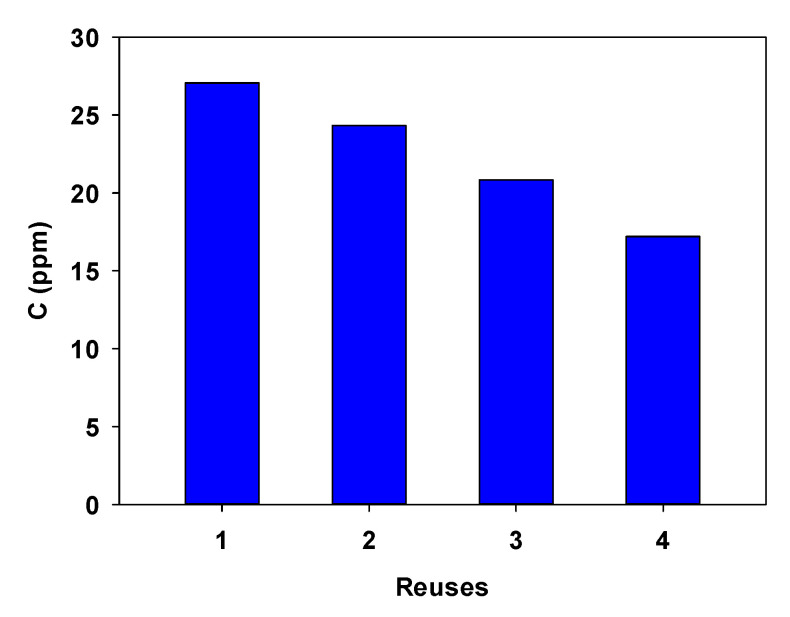
Reuse of HT-DSC for the adsorption of 4-NPhOH. Experimental conditions: 25 mL of adsorbate solution, 100 mg of sorbent, and 22 °C. Where “1” is fresh material.

Three reuses were carried with HT-DSC as adsorbent with a 4-NPhOH concentration of 30 ppm. Figure 6 shows the adsorption cycles performed at 30 min. As can be seen, the recyclability of HT-DSC is effective, showing an adsorption capacity similar to that obtained by the fresh adsorbent. Only a 10% decrease in adsorption capacity was observed for each recycle. A washing step with CH_3_OH was carried out between each reuse to desorb the adsorbed 4-NPhOH on the HT-DSC. It is known that the hydrophobic interaction between the aromatic ring of 4-NPhOH and the hydrocarbon portion of deoxycholate molecules on the material produces weak bonds, which are easily broken out during the washing step with CH_3_OH.

## 4. Conclusions

In this work, we used a hydrotalcite intercalated with deoxycholate anion as sorbent to remove the organic pollutant nonylphenol from water. Although the adsorption rate was influenced by the pollutant concentration, the adsorption process reached equilibrium after about 60 min. The ratio of the number of sorbent sites to that of nonylphenol molecules increased with decreasing concentration of pollutant in the aqueous solution. The pollutant was very rapidly adsorbed onto the outer surface of the hydrotalcite. 4-NPhOH was chemisorbed onto the hydrophobic, hydrocarbon portion of deoxycholate on the outer surface of the hydrotalcite. Once the outer surface saturated, however, the adsorbate diffused over the pore network of the solid and was adsorbed on the interlayer region, albeit more slowly than it was on the solid surface. Nonylphenol adsorption onto the hydrotalcite was found to be a pseudo-second order process. The results suggest that the Freundlich model describes the sorption equilibrium proving a chemisorption interaction. Finally, for 4-NPhOH, a value of R_L_ means that the adsorption process is favorable.

## Figures and Tables

**Table 1:** Compositional and structural properties determined for HT-DSC [31].

Solid	Mg/Al_Ther_	Mg/Al_exp_^a^	A (nm)	C (nm)
Hydrotalcite	2.5	2.41	0.305	9.810

^a^ Mg/Alexp ratio was calculated by inductively coupled plasma mass spectrometry (ICP-MS).

**Table 2:** Kinetics parameters and correlation coefficients for the sorbent.

	T (K)	Pseudo-First Order		Pseudo-Second Order		Elovich		Intra-Particle Diffusion		
		R^2^	K_1_	R^2^	K_2_	R^2^	B	R^2^	K_Intra_	c
30	298	0.824	0.010	0.938	0.169	0.960	3.917	0.881	0.077	4.176
25	298	0.962	0.009	0.980	0.154	0.961	2.699	0.910	0.117	3.426
20	298	0.921	0.031	0.993	0.148	0.910	2.075	0.712	0.138	2.828
15	298	0.893	0.051	0.957	0.132	0.802	1.962	0.559	0.127	1.810

**Table 3:** Isotherm adsorption parameters for the different models.

**Langmuir**	Q = 53.19	b = 5 · 10^−3^	R^2^ = 0.956
**Freundlich**	K_F_ = 0.306	n = 1.079	R^2^ = 0.999
**Temkin**	K_T_ = 0.488	b_T_ = 2.919	R^2^ = 0.998

## Data Availability

The authors declare that the manuscript is original, has not been submitted to or published in any other journal, and the data in the manuscript is real.

## Supplementary Materials

The following supporting information can be downloaded at: https://www.mdpi.com/article/10.3390/ijerph19127214/s1, Figure S1: Kinetic models for 15 ppm concentration: (a) pseudo-first-order kinetic model, (b) pseudo-second-order kinetic model, (c) Elovich kinetic model, (d) and interparticle diffusion kinetic model. Figure S2: Kinetic models for 25 ppm concentration: (a) pseudo-first-order kinetic model, (b) pseudo-second-order kinetic model, (c) Elovich kinetic model, (d) and interparticle diffusion kinetic model. Figure S3: Kinetic models for 30 ppm concentration: (a) pseudo-first-order kinetic model, (b) pseudo-second-order kinetic model, (c) Elovich kinetic model, (d) and interparticle diffusion kinetic model.
