# Supplementary material for: Efficient Removal of Nonylphenol Isomers from Water by Use of Organo-Hydrotalcites
Source: Int J Environ Res Public Health. 2022 Jun 12;19(12):7214. doi: 10.3390/ijerph19127214 (PMC9222827; doi:10.3390/ijerph19127214)

# SUPPLEMENTARY INFORMATION

## Efficient removal of nonylphenol isomers from water by use of organo-hydrotalcites

Daniel Cosano*, Dolores Esquivel, Francisco J. Romero-Salguero, César Jiménez-Sanchidrián and José Rafael Ruiz*

*Departamento de Química Orgánica, Instituto Universitario de Investigación en Química Fina y Nanoquímica IUIQFN, Facultad de Ciencias, Universidad de Córdoba, Campus de Rabanales, Edificio Marie Curie, E-14071 Córdoba, Spain*

*Corresponding authors. E-mail address: q92cohid@uco.es (D. Cosano); qo1ruarj@uco.es (J.R. Ruiz), Tel.: 34 957218638; Fax: 34 957212066.

**Figure S1.** Kinetic models for 15 ppm concentration: a) Pseudo-first-order kinetic model, b) Pseudo-second-order kinetic model, c) Elovich kinetic model, d) and Interparticle diffusion kinetic model.

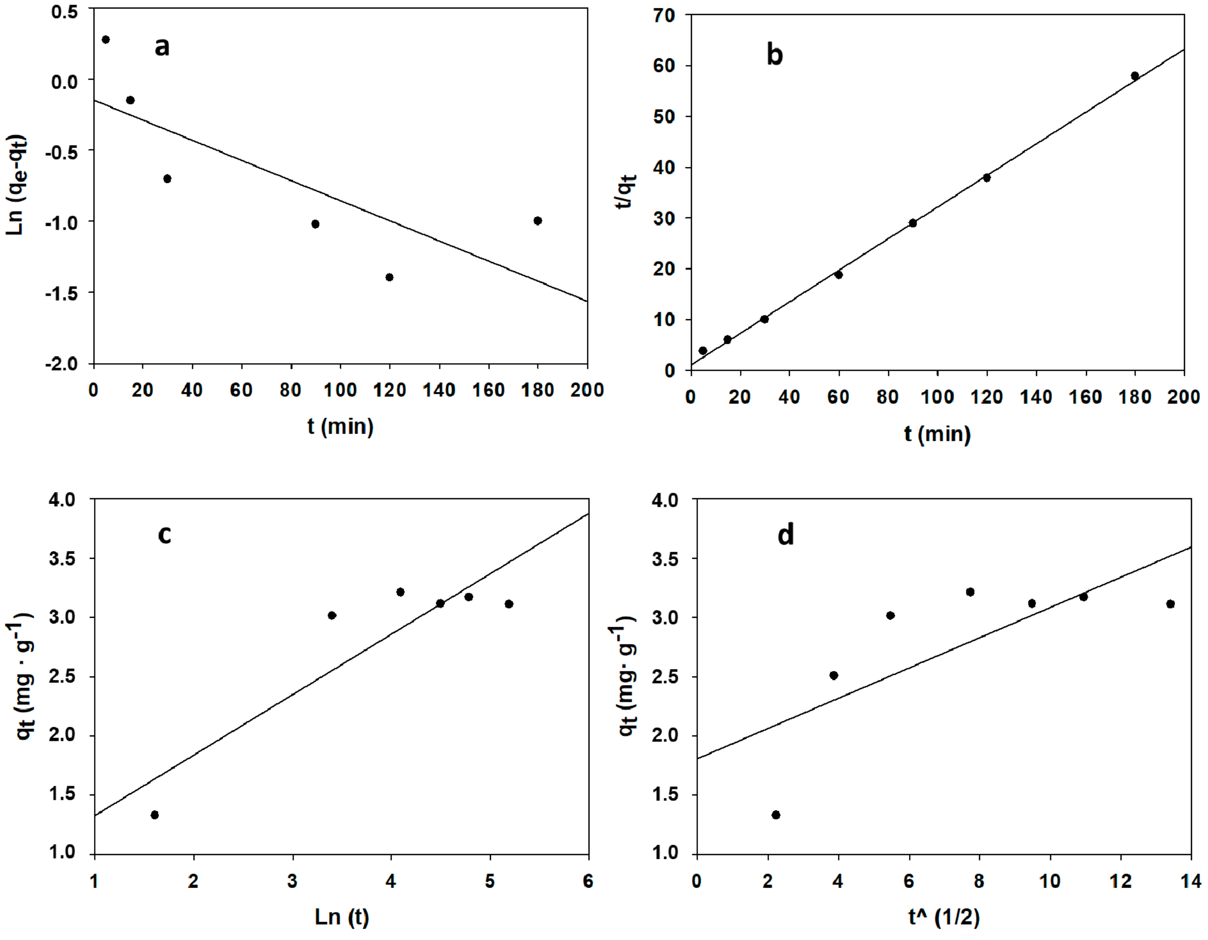

**Figure S2.** Kinetic models for 25 ppm concentration: a) Pseudo-first-order kinetic model, b) Pseudo-second-order kinetic model, c) Elovich kinetic model, d) and Interparticle diffusion kinetic model.

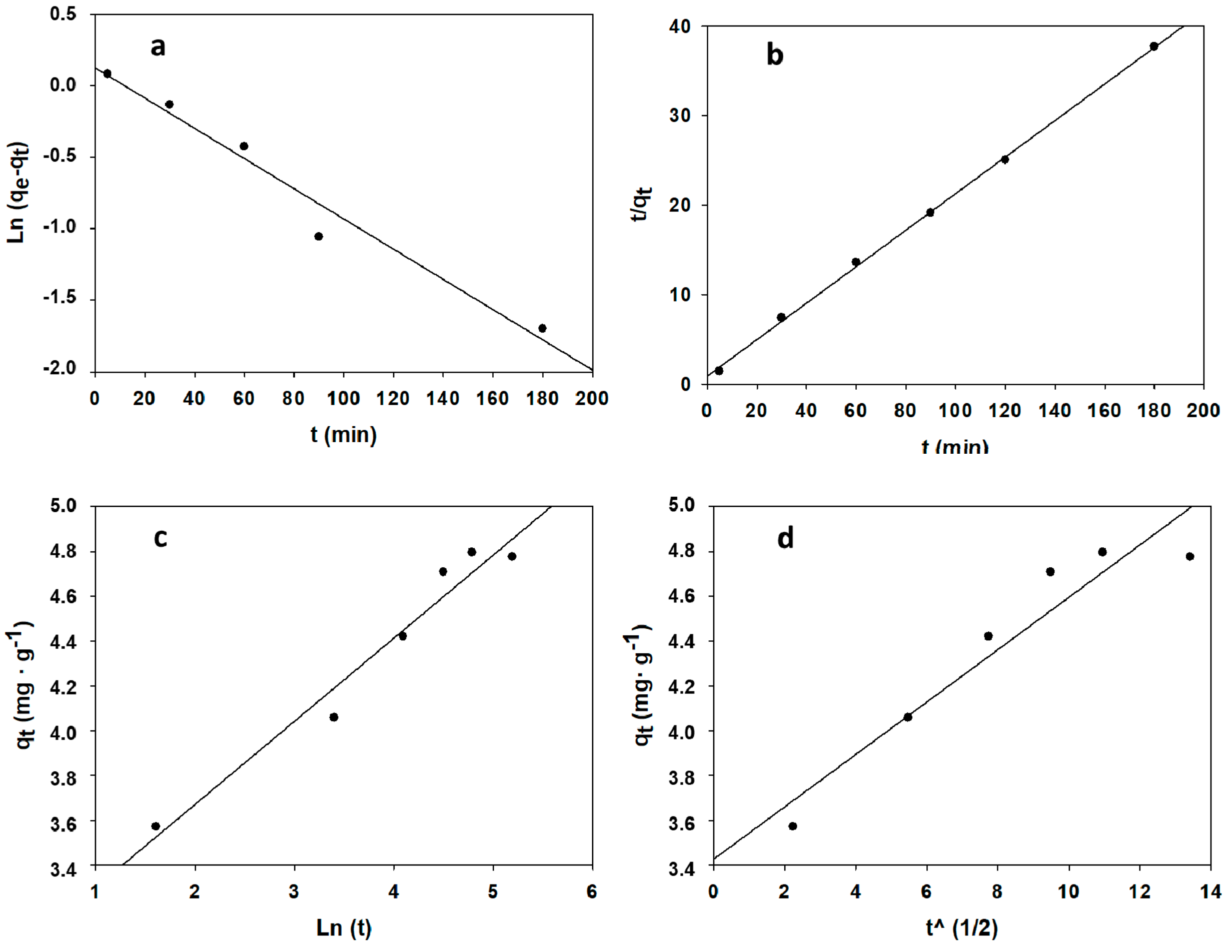

**Figure S3.** Kinetic models for 30 ppm concentration: a) Pseudo-first-order kinetic model, b) Pseudo-second-order kinetic model, c) Elovich kinetic model, d) and Interparticle diffusion kinetic model.

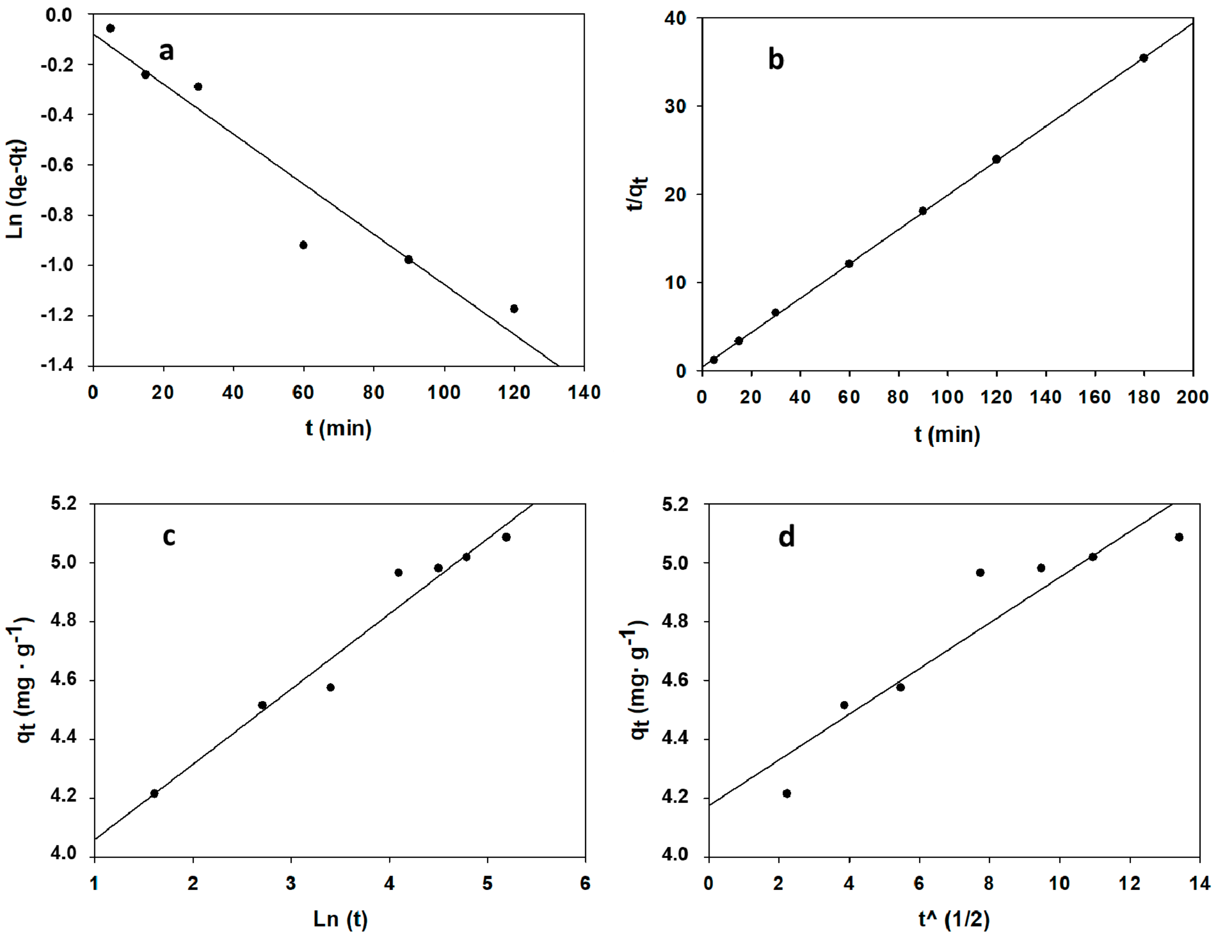

Supplement: Supplementary file 1 [file ijerph-19-07214-s001.zip › ijerph-1691689-supplementary.pdf]
